# Joint analysis of case-parents trio and unrelated case-control designs in large scale association studies

**DOI:** 10.1186/1753-6561-1-s1-s28

**Published:** 2007-12-18

**Authors:** Jungnam Joo, Xin Tian, Gang Zheng, Mario Stylianou, Jing-Ping Lin, Nancy L Geller

**Affiliations:** 1 Office of Biostatistics Research, National Heart, Lung and Blood Institute, 6701 Rockledge Drive, MSC 7913, Bethesda, MD 20892-7913, USA

## Abstract

We present a new method for testing association when data from both case-parents trios and unrelated controls are available. Our method combines test statistics for case-parents trio and unrelated case-control studies by adjusting for the correlation that arises when the same set of cases is used for both tests. We further consider several analytical approaches for two-stage studies on a large number of markers, including methods based on the joint analysis. The performance of the proposed approaches is examined by analyzing the simulated data provided by the Genetic Analysis Workshop 15.

## Background

Genetic association studies are a popular method to detect genetic markers associated with a complex human disease. Two common designs in genetic association studies are family-based designs using case-parents trios and population-based designs using unrelated cases and controls. The transmission disequilibrium test (TDT) is frequently used to analyze the case-parents trio data [1]. The TDT tests for both linkage and association and is not sensitive to population admixture and stratification. Using a likelihood approach, Schaid and Sommer [2] proposed TDT-type statistics that are more powerful than the TDT for a specific genetic model (see also [3]). For the unrelated case-control design, a linear trend test [4], which is often more powerful than the TDT based on case-parents trios, can be considered specifically when obtaining a sufficient number of trios is difficult.

Data that contain both case-parents trios and unrelated cases and controls on the same set of markers are increasingly available. Nagelkerke et al. [5] provided a few situations where such a mixture of case-parents trios and unrelated cases and controls can occur: 1) a case-parents trio design was originally considered and then unrelated controls were added, 2) a case-control design was originally considered and then the parents of the cases were added to confirm the findings. Such designs are typically analyzed in two stages, and strategies for analyzing this type of data while fully utilizing the given information are important.

In this paper, we study several approaches for testing genome-wide association in such situations. Based on the design, either a TDT-type statistic or a linear trend test will be used in the first stage to select a proportion of markers that will be tested in the second stage. The other test will then be applied in the second stage while controlling the genome-wide false positive rates by adjusting for the correlation with the first stage. Following a recently proposed method by Skol et al. [6], we also study a joint analysis for the second stage.

## Methods

Consider a marker with two alleles, M and N, where M itself is a risk allele or is in linkage disequilibrium with a risk allele with frequency *p*, and N is a normal allele with frequency *q *= 1 - *p*. Penetrances are defined as the probabilities of disease conditional on the genotypes, that is, *f*_0 _= Pr(disease|NN), *f*_1 _= Pr(disease|NM), and *f*_2 _= Pr(disease|MM). No association implies *f*_0 _= *f*_1 _= *f*_2_, whereas *f*_0 _≤ *f*_1 _≤ *f*_2 _with at least one strict inequality implies there is an association between the marker and a disease. Using *f*_0 _as a baseline penetrance, the genotype relative risks are defined as *ψ*_*i *_= *f*_*i*_/*f*_0 _for *i *= 1, 2. A genetic model is recessive, additive, or dominant when *f*_0 _= *f*_1 _(or *ψ*_1 _= 1, *ψ*_2 _= *ψ*), *f*_1 _= (*f*_0 _+ *f*_2_)/2 (or *ψ*_1 _= *ψ*, *ψ*_2 _= 2*ψ*-1), or *f*_1 _= *f*_2 _(or *ψ*_1 _= *ψ*_2 _= *ψ*).

### Case-parents trio design

In the case-parents trio design, cases and their parents are selected from the population and their genotypes are obtained. There are six possible parental mating types for a marker with two alleles M and N: 1) MM × MM, 2) MM × NM, 3) MM × NN, 4) NM × NM, 5) NM × NN, and 6) NN × NN. These six mating types are given in the first column of Table 1. The second column provides case genotypes for each mating type, and the third column is the sample size of trios under each mating type. The probabilities of parental mating types can be calculated by assuming Hardy-Weinberg equilibrium (HWE), and the probability of each *n*_*ij *_can then be obtained and is presented in the fourth column. The last column contains the probabilities of a case genotype given parental mating type (Schaid and Sommer [2]).

**Table 1:** Conditional probabilities of genotype given parental mating types and offspring disease status

Parental mating type	Case genotype	Count	Probability of trio	Conditional probability
1) MM × MM	MM	*n*_12_	*p*^4^*ψ*_2_/*T*	1
				
2) MM × NM	MM	*n*_22_	2*p*^3^*q*(*ψ*_1 _+ *ψ*_2_)/*T*	*ψ*_2_/(*ψ*_1 _+ *ψ*_2_)
	NM	*n*_21_		*ψ*_1_/(*ψ*_1 _+ *ψ*_2_)
				
3) MM × NN	NM	*n*_31_	2*p*^2^*q*^2^*ψ*_1_/*T*	1
				
4) NM × NM	MM	*n*_42_	2*p*^2^*q*^2^(*ψ*_2 _+ 2*ψ*_1 _+ 1)/*T*	*ψ*_2_/(*ψ*_2 _+ 2*ψ*_1 _+ 1)
	NM	*n*_41_		2*ψ*_1_/(*ψ*_2 _+ 2*ψ*_1 _+ 1)
	NN	*n*_40_		1/(*ψ*_2 _+ 2*ψ*_1 _+ 1)
				
5) NM × NN	NM	*n*_51_	2*pq*^3^(*ψ*_1 _+ 1)/*T*	*ψ*_1_/(*ψ*_1 _+ 1)
	NN	*n*_50_		1/(*ψ*_1 _+ 1)
				
6) NN × NN	NN	*n*_60_	*q*^4^1/*T*	1

*T *= *ψ*_2_*p*^2 ^+ *ψ*_1_2*pq *+ *q*^2^

Schaid and Sommer [2] suggested an analysis conditional on parental mating types that provides unbiased estimates of genotype relative risks. Denote the likelihood function for a given model as L(*ψ*), then the score test for *H*_0_: *ψ *= 1 can be obtained by ∂logL(*ψ*)/∂*ψ*/{-∂^2^logL(*ψ*)/∂*ψ*^2^}^1/2^|_*ψ *= 1_.

### Unrelated case-control design

For the unrelated case-control design, denote the genotype counts of three genotypes NN, MN and MM as (*r*_0_, *r*_1_, *r*_2_) in cases and (*s*_0_, *s*_1_, *s*_2_) in controls that follow multinomial distributions *mul*(*R: p*_0_, *p*_1_, *p*_2_) and *mul*(*S: q*_0_, *q*_1_, *q*_2_). Then the null hypothesis of no association implies *p*_*i *_= *q*_*i *_for each *i*.

Sasieni [4] proposed a method that uses the marker genotype as a covariate in the logistic regression model where the genotype is coded by increasing scores, that is, 0, *x*, and 1 for NN, NM, and MM, where 0 ≤ *x *≤ 1. The optimal scores for recessive, additive and dominant models are *x *= 0, 1/2, and 1 [4,7] and the trend test [7] is given by $Z_{CC}=\frac{U(x)}{\sqrt{Var(U(x))}}$, where $U(x)=\sum_{i=0}^{2}x_{i}(1-R/N)r_{i}-\sum_{i=0}^{2}x_{i}(R/N)s_{i}$, and $Var(U(x))=N^{-1}RS\{\sum_{i}x_{i}^{2}p_{i}-{\left(\sum_{i}x_{i}p_{i}\right)}^{2}\}$ for (*x*_0_, *x*_1_, *x*_2_) = (0, *x*, 1) and *N *= *R*+*S*. Under the null hypothesis, *Z*_*CC *_asymptotically follows the standard normal distribution.

### Combined test of *Z*_*TDT *_and *Z*_*CC*_

Because the cases used in *Z*_*TDT *_and *Z*_*CC *_overlap, results from the two tests are correlated, and this correlation, *ρ*, must be considered when obtaining a combined test. By noting that both tests are functions of a multinomial random variable *n *with dimension 10 for the 10 *n*_*ij *_categories from Table 1, the correlation between *Z*_*TDT *_and *Z*_*CC *_can be obtained given a specific genetic model (Appendix). The probability of each category can be consistently estimated by the observed counts and *ρ *can be consistently estimated by the sample correlation between *Z*_*TDT *_and *Z*_*CC*_.

We propose the weighted average, $Z_{\text{joint}}=\frac{\sqrt{w_{1}}Z_{TDT}+\sqrt{w_{2}}Z_{CC}}{\sqrt{\left(w_{1}+w_{2}+2\sqrt{w_{1}w_{2}}\rho \right)}}$, as a test statistic in a joint analysis. We consider a uniform weight, that is, *w*_1 _= *w*_2 _= 1 [8,9] for simplicity. Other choices of weight, such as a weight proportional to the number of informative cases used in each test, can also be considered.

### Two-stage method in large scale association studies

To test *K *markers in a two-stage analysis, we consider four strategies that use either *Z*_*TDT *_or *Z*_*CC *_in the first stage based on the intended design, and in each situation, the other test or the joint test is applied in the second stage. As in Skol et al. [6], we obtain thresholds *C*_1 _and *C*_2 _(or *C*_joint_) for two stages in each strategy by controlling the genome-wide significance level at *α*. *C*_1 _can be obtained as *C*_1 _= Φ^-1^(1 - *π*_1_/2), where *π*_1 _is the proportion of markers selected in the first stage. On the other hand, *C*_joint _and *C*_2 _need to be calculated iteratively so that they satisfy

$$\sum_{i=1}^{K}P(|Z_{1i}|>C_{1},|Z_{jointi}|>C_{joint})=\alpha$$

or

$$\sum_{i=1}^{K}P(|Z_{1i}|>C_{1},|Z_{2i}|>C_{2},Z_{1i}Z_{2i}>0)=\alpha$$

when the joint analysis is used or when the other test is used. Here, *Z*_1*i *_and *Z*_2*i *_denote the tests used in the first and the second stage for the *i*^th ^SNP (*Z*_2*i *_is replaced by *Z*_*jointi *_when the joint analysis is used in the second stage). We need the subscript *i *because the correlations between two tests for different SNPs are generally not the same. Under HWE, however, we can show this correlation is a constant (Appendix), and these equations can then be simplified to *P*(|*Z*_1_| > *C*_1_, |*Z*_joint_| > *C*_joint_) = *α*/*K *and *P*(|*Z*_1_| > *C*_1_, |*Z*_2_| > *C*_2_, *Z*_1_*Z*_2 _> 0) = *α*/*K*.

### Data

The Genetic Analysis Workshop 15 provided simulated rheumatoid arthritis data that contain 1500 families with affected sib pairs and their parents, and 2000 unrelated controls on 9187 SNPs distributed throughout the genome. We used the first simulated data set and we randomly selected one from the affected sib pairs for data analysis. The minor allele frequencies of all 9187 SNPs were greater than 1%.

## Results

To apply the two-stage analysis, we first obtained the threshold for each strategy using *π*_1 _= 0.1 (*C*_1 _= 1.6449) and Eq. (1) and (2). Therefore, we control the genome-wide false-positive rate at 0.05, and we define a "significant" SNP as one with test statistic greater than the threshold in both stages. As expected, a slightly larger threshold for the second stage is required for the joint analysis to control the same genome-wide false-positive rate (*C*_2 _= 4.5121 vs. *C*_joint _= 4.5470) [6]. Table 2 summarizes results based on an additive genetic model. The chromosome, SNP name, and distance from the nearest major gene are listed in the first three columns of the table, and if the SNP was selected by the specified method (last three columns), the *p*-value from the second stage is listed. We noticed that even with a larger threshold required, the joint analysis in the second stage found more significant SNPs near the major genes. Also, we noticed that when the joint analysis was used in the second stage, the same set of significant SNPs was found regardless of the choice of the test statistic in the first stage. However, different results were obtained when either *Z*_*TDT *_or *Z*_*CC *_was used in the first stage followed by the other test in the second stage. Specifically, the joint analysis using either *Z*_*TDT *_or *Z*_*CC *_in the first stage found 18 significant SNPs among which 9 and 14 were located within 1 Mb (bold) and 5 Mb (italic) of the major genes. When *Z*_*TDT *_in the first stage was followed by *Z*_*CC *_in the second stage, we found 17 significant SNPs, and 8 of these were located within 1 Mb of the causal genes, and 13 were located within 5 Mb. These methods found SNPs near the major genes on chromosome 6, 11, and 18. On the other hand, when we used *Z*_*CC *_in the first stage followed by *Z*_*TDT *_in the second stage, a total of 10 significant SNPs were found: 7 of them were located within 1 Mb of a major gene only on chromosome 6 and 11, and 10 were located within 5 Mb. A SNP near the major gene on chromosome 18 was not found by this method.

**Table 2:** Two-stage analysis: selected SNPs and their corresponding *p*-values in the second stage

Chromosome	SNP	Distance (Mb)	*Z*_*TDT *_then *Z*_*CC*_	*Z*_*CC *_then *Z*_*TDT*_	*Z*_*TDT *_or *Z*_*CC *_then *Z*_joint_
6	SNP6_128	7.13	1.02 × 10^-8^		1.61 × 10^-7^
	SNP6_129	7.10	4.01 × 10^-13^		1.45 × 10^-8^
	SNP6_130	7.10	1.48 × 10^-13^		1.28 × 10^-9^
	SNP6_134	6.41	5.76 × 10^-10^		1.33 × 10^-^6
	*SNP6_138*^b^	3.73	*2.89 × 10*^-15^	*2.85 × 10*^-7^	*1.07 × 10*^-13^
	*SNP6_139*	3.72	*2.44 × 10*^-15^	*3.75 × 10*^-7^	*1.22 × 10*^-13^
	*SNP6_145*	2.92	*1.03 × 10*^-9^	*5.20 × 10*^-6^	*1.24 × 10*^-9^
	*SNP6_147*	2.22	*1.37 × 10*^-8^		*3.05 × 10*^-7^
	*SNP6_150*	1.39	*5.99 × 10*^-7^		*6.25 × 10*^-7^
	**SNP6_152**^c^	0.04	*2.38 × 10*^-221^	*1.55 × 10*^-94^	*4.31 × 10*^-193^
	**SNP6_153**	0.01	0*^a^	*7.33 × 10*^-206^	0*^a^
	**SNP6_154**	0.04	0*	*1.23 × 10*^-182^	0*
	**SNP6_155**	0.29	*4.49 × 10*^-87^	*1.91 × 10*^-49^	*7.36 × 10*^-86^
	**SNP6_160**	0.65	*8.62 × 19*^-10^	*8.20 × 10*^-9^	*1.16 × 10*^-11^
	**SNP6_162**	0.13	*9.15 × 10*^-24^	*1.78 × 10*^-15^	*9.36 × 10*^-26^
					
11	SNP11_387	0.19			*3.00 × 10*^-6^
	SNP11_389	0.03	*2.78 × 10*^-28^	*3.33 × 10*^-15^	*2.41 × 10*^-27^
18	SNP18_269	0.02	*2.25 × 10*^-8^		*1.56 × 10*^-8^

^a^0*, *p*-value < 10^-300^. Test statistics are based on an additive genetic model.

^b^Italic, SNPs within 5 Mb of the major genes.

^c^Bold, SNPs within 1 Mb of the major genes.

When we applied these three tests (*Z*_*CC*_, *Z*_*TDT*_, *Z*_joint_) to a single-stage analysis, these tests found the same set of SNPs identified in a two-stage analysis with the corresponding test at the second stage. That is, *Z*_*CC*_, *Z*_*TDT*_, *Z*_jointi _in a single-stage found 17, 10, and 18 SNPs in columns 4, 5, and 6 of Table 2. This implies that a two-stage analysis can maintain power with a substantially reduced genotyping cost while controlling the same genome-wide false-positive rate [6].

## Discussion

In this paper, we presented a new method for testing association when both case-parents trios and unrelated controls are available. Because parents are selected for having an affected child, we consider the characteristics of non-affected parents to be different from those of unrelated controls in case-control studies. Thus, the genotype information of parents was used only for *Z*_*TDT *_and not for *Z*_*CC*_. By adjusting for the correlation between the two test statistics (*Z*_*TDT *_and *Z*_*CC*_), we proposed a combined test statistic for analyzing such data.

For data with a large number of markers in a two-stage analysis, we considered several analytical approaches following the method by Skol et al. [6]. Even with a slightly larger threshold required, more SNPs near the major genes were found using the joint analysis in the second stage. Also, we noticed the choice of test for the first stage was important when two separate tests were used in the two stages, but when the joint analysis was used, the impact of which test was used first seemed to be less important. The added benefit of the joint analysis was rather minor compared to what was studied by Skol et al. [6] because the two tests for the first and the second stages were highly correlated even without using the joint analysis. Nevertheless, the joint analysis found slightly more significant SNPs and is robust against the choice of the first stage test. These properties suggest that the joint analysis would be desirable.

Our method can be generalized to data with missing genotypes by either imputing the missing genotypes based on partially available data [5,10], or by omitting cases without complete parental information from *Z*_*TDT*_. In this situation, the correlation between *Z*_*TDT *_and *Z*_*CC *_will decrease, and therefore, the advantage of the joint analysis could be accentuated. Complete justification, however, requires further study.

## Conclusion

We presented a new method for testing association when data from both case-parents trios and unrelated controls are available. By deriving the correlation of test statistics for these two designs, we proposed a combined test as a joint analysis. In a two-stage analysis for testing a large number of markers, we found that the joint analysis detects more SNPs near the major genes than other methods that do not use the combined test in the second stage. This approach is also robust against the choice of the first stage test.

## Appendix

When the conditional likelihood is used for *Z*_*TDT*_, *n*_1 _= *n*_12_, *n*_2 _= (*n*_21_, *n*_22_), *n*_3 _= *n*_31_, *n*_4 _= (*n*_40_, *n*_41_, *n*_42_), *n*_5 _= (*n*_50_, *n*_51_), and *n*_6 _= *n*_60 _are independent random variables conditional on parental mating types (*m*) where *n*_2 _and *n*_5 _follow a binomial distribution and *n*_4 _follows a trinomial distribution with probabilities given in column 5 of Table 1[2]. The score test for *H*_0_: *ψ *= 1 is then written as $Z_{TDT}=\frac{U_{T}(n)-E(U_{T}(n)|m)}{\sqrt{{\text{Var(U}}_{\text{T}}\text{(n)|}m\text{)}}}$, where *U*_*T*_(*n*) = *n*_22_+*n*_42_, *n*_22_+*n*_42_+0.5(*n*_21_+*n*_41_+*n*_51_) and *n*_42_+*n*_41_+*n*_51 _for the recessive, additive, and dominant models. By applying the variance decomposition formula, we obtain the correlation between *Z*_*TDT *_and *Z*_*CC *_as $(1-R/N)\frac{E(\sqrt{{\text{Var(U}}_{\text{T}}\text{(n)|}m\text{)}})}{\sqrt{\text{Var(U(x))}}}$. An additional distributional assumption needs to be made for parental genotypes. We considered six parental mating types as a six dimensional multinomial distribution, and the corresponding probabilities were consistently estimated by the observed counts.

Under HWE, we can show that the correlation for three models can be simplified to $\sqrt{1-R/N}$ when all cases have parental genotypes available. When only a proportion of cases overlaps between case-parents and case-control designs, we can introduce an additional parameter *η *< 1 such that $\sum_{ij}n_{ij}=\eta R$, and the correlation between *Z*_*TDT *_and *Z*_*CC *_is reduced to $\eta \sqrt{1-R/N}$.

## Competing interests

The author(s) declare that they have no competing interests.
